# Investigating the Cellular Responses to Combined Nisin and Urolithin B Treatment (7:3) in HKB-11 Lymphoma Cells

**DOI:** 10.3390/ijms26157369

**Published:** 2025-07-30

**Authors:** Ahmad K. Al-Khazaleh, Muhammad A. Alsherbiny, Dennis Chang, Gerald Münch, Deep Jyoti Bhuyan

**Affiliations:** 1 NICM Health Research Institute, Western Sydney University, Penrith, NSW 2751, Australia; 19316068@student.westernsydney.edu.au (A.K.A.-K.); d.chang@westernsydney.edu.au (D.C.); 2 Pharmacognosy Department, Faculty of Pharmacy, Cairo University, Cairo 11562, Egypt; muhammad.alsherbiny@pharma.cu.edu.eg; 3 Freedman Foundation Metabolomics Facility, Innovation Centre, Victor Chang Cardiac Research Institute, Sydney, NSW 2010, Australia; 4 Pharmacology Unit, School of Medicine, Western Sydney University, Campbelltown, NSW 2560, Australia; g.muench@westernsydney.edu.au; 5 School of Science, Western Sydney University, Penrith, NSW 2751, Australia

**Keywords:** lymphoma, postbiotics, nisin, urolithin B, synergy, proteomics, apoptosis

## Abstract

Lymphoma continues to pose a serious challenge to global health, underscoring the urgent need for new therapeutic strategies. Recently, the gut microbiome has been shown to play a potential role in regulating immune responses and influencing cancer progression. However, its molecular mechanisms of action in lymphoma remain poorly understood. This study investigates the antiproliferative and apoptotic activities of gut microbiota-derived metabolites, specifically nisin (N) and urolithin B (UB), individually and in combination 7:3 (5750 μM), against the human lymphoma cell line HKB-11. Comprehensive evaluations were performed using Alamar Blue viability assays, combination index (CI) analyses, reactive oxygen species (ROS) quantification, flow cytometry for apoptosis detection, and advanced bottom-up proteomics analyses. N and UB exhibited potent antiproliferative activity, with the 7:3 combination demonstrating strong synergistic effects (CI < 1), significantly enhancing apoptosis (*p* < 0.01) and ROS production (*p* < 0.0001) compared to the untreated control. Proteomics analyses revealed substantial alterations in proteins crucial to ribosomal biogenesis, mitochondrial function, cell cycle control, and apoptosis regulation, including a marked downregulation of ribosomal proteins (RPS27; Log_2_FC = −3.47) and UBE2N (Log_2_FC = −0.60). These findings highlight the potential of N and UB combinations as a novel and practical therapeutic approach for lymphoma treatment, warranting further in vivo exploration and clinical validation.

## 1. Introduction

Lymphoma encompasses many haematological malignancies arising from B, T, or natural killer (NK) lymphocytes. It is clinically classified into two main types: Hodgkin’s lymphoma (HL) and non-Hodgkin lymphoma (NHL). NHL accounts for over 85% of all lymphoma cases and includes highly aggressive subtypes such as Burkitt lymphoma and diffuse large B-cell lymphoma (DLBCL). These forms are often characterised by rapid proliferation, treatment resistance, and poor outcomes, particularly in relapsed or refractory stages [1,2]. Despite the progress achieved with immunochemotherapy and targeted agents, long-term survival remains suboptimal in many patients. Thus, the failure of standard therapies to effectively control disease progression, especially in aggressive lymphoma subtypes, highlights the need for novel and safer therapeutic alternatives that can complement or enhance current treatment regimens.

Recent research has revealed the crucial role of the gut microbiome in regulating cancer progression, immune surveillance, and response to therapy [3,4,5]. The gut microbiota generates a wide range of bioactive metabolites collectively termed postbiotics that can impact host physiology and disease outcomes. These metabolites include short-chain fatty acids (SCFAs), polyamines, indoles, and microbial conversion products such as urolithins and bacteriocins. Many of these compounds exert anticancer effects through mechanisms involving immune activation, apoptosis induction, cell cycle arrest, and epigenetic regulation [6,7,8].

In preclinical studies, the postbiotics nisin (N) and urolithin B (UB) have demonstrated potential anticancer properties. N is a lantibiotic produced by *Lactococcus lactis* [3]. It is a membrane-active compound that forms pores in bacterial membranes. In cancer cells such as MCF7 and MDA-MB-231 breast adenocarcinoma cell lines, N has been reported to disrupt membrane integrity, modulate reactive oxygen species (ROS), trigger mitochondrial dysfunction, and induce apoptosis [9]. It also modulates immune responses and has been shown to inhibit tumour growth in models of head and neck squamous cell carcinoma, colorectal cancer, and leukemia [10,11].

UB is derived from the gut microbial metabolism of ellagitannins, a type of polyphenolic compound found in foods such as pomegranate, berries, and nuts. Unlike urolithin A, UB showed distinct pharmacological profiles, including antioxidant, anti-inflammatory, and antiproliferative effects [3]. Moreover, it has been reported to suppress tumour growth in prostate, breast, and colon cancer by modulating signalling pathways such as NF-κB, MAPK, and PI3K/AKT [12,13]. UB also affects the tumour microenvironment by regulating cytokine release and promoting immune-mediated cytotoxicity [14]. While the individual effects of N and UB have been explored against cancer, the potential synergy between these two postbiotics in cancer treatment has not been extensively investigated [3,15]. A combination approach may offer enhanced efficacy by simultaneously targeting multiple pathways, including membrane disruption, oxidative stress, immune modulation, signal transduction inhibition, and their complementary mechanisms. N, acting at the cell surface, and UB, modulating intracellular pathways, suggest the potential for improved anti-tumour activity with minimal systemic toxicity [16].

Although both N and UB are individually known for their anticancer activities [9,13,17], their combined effects and mechanisms have not been thoroughly explored, particularly in lymphoma. Combining these two agents leverages complementary mechanisms, namely nisin’s membrane-disrupting, ROS-mediated pathways and urolithin B’s mitochondrial and epigenetic apoptosis pathways, which may yield synergistic activity while minimising off-target effects [7,16]. Furthermore, both compounds are naturally derived, with well-established safety profiles and translational potential, making them more practical candidates than less-characterised analogues or derivatives. This study aims to evaluate the combination effects of these agents and elucidate the underlying molecular mechanisms.

This study investigates the combined antiproliferative effects of N and UB in cellular models of lymphoma. Furthermore, it evaluates their effects on cell viability, apoptosis, and protein expression profiles, aiming to uncover underlying mechanisms and support the development of postbiotic-based combination therapies in the future.

## 2. Results and Discussion

### 2.1. The Antiproliferative Activity of the Postbiotics (N and UB), and Their Synergistic Combination (7:3) Against the HKB-11 (BL) Human Cell Line

We recently evaluated nine different ratios of N and UB against the HKB-11 lymphoma cells [18]. Strong synergistic interactions were observed between N and UB at combination 4:6 and 7:3 (5600 μM N: 150 μM UB), with CI values <1 compared to the mono treatments of N and UB [18]. This study assessed the antiproliferative potential and apoptotic effects of the combination at a 7:3 ratio in two human lymphoma cell lines (HKB-11 and Hs 313.T) and a normal stromal cell line (HS-5) to determine efficacy, selectivity, and the therapeutic window (Table 1).

The selected concentrations of N and UB were determined empirically through serial titration experiments to achieve measurable antiproliferative and apoptotic effects while maintaining selectivity over normal stromal cells [18]. High micromolar-to-millimolar concentrations are not uncommon for natural products in vitro, given their limited cell permeability and serum binding [12,13]. In contrast, doxorubicin is a synthetic chemotherapeutic with significantly higher potency and a distinct mechanism of action [12,13,19]; therefore, the two are not directly comparable.

The N and UB combination (7:3) showed an effect on both lymphoma cell lines. At the highest tested concentration of 5750 µM, complete cell growth inhibition was observed, reaching 100.16 ± 0.02% for HKB-11 and 100.45 ± 0.11% for Hs 313.T. Similarly, near-complete cell growth inhibition persisted at 2875 µM (100.11 ± 0.03% for HKB-11; 99.05 ± 1.16% for Hs 313.T).

Hs 313.T cells were more sensitive to the combination, with a notably lower IC_50_ of 332.2 µM compared to the HKB-11 cells (IC_50_ = 820 µM). Specifically, at intermediate concentrations such as 718.75 µM and 359.38 µM, significantly greater growth inhibition was observed for Hs 313.T cells (82.71 ± 0.88% and 56.49 ± 3.83%, respectively) compared to HKB-11 cells (49.15 ± 7.03% and 14.40 ± 5.52%, respectively; *p* < 0.05; Table 1). These differences reflect a clear differential response pattern between the two lymphoma cell lines, highlighting Hs 313 T’s consistently greater susceptibility at lower and intermediate doses. The impact of the N and B combination on HS-5 normal stromal cells indicated a favourable therapeutic window. At lower concentrations (359.38 µM and below), the viability of HS-5 cells exceeded 90%, suggesting minimal off-target toxicity. Even at intermediate doses, such as 718.75 µM, stromal viability remained relatively high (72.96 ± 9.29%) while effectively targeting lymphoma cells, further underscoring the therapeutic potential of this approach.

The data confirmed a selective cytotoxicity profile of the N:UB 7:3 combination against lymphoma cell lines. Hs 313.T’s greater sensitivity at intermediate doses highlighted potential genetic or metabolic vulnerabilities distinct from HKB-11. HKB-11 exhibited pronounced sensitivity at maximal concentrations, which could reflect aggressive proliferative traits due to its hybrid Burkitt lymphoma origin. Such differential sensitivity highlights inherent biological differences between lymphoma subtypes, underscoring the need for targeted therapeutic approaches. HKB-11 cells were selected for further molecular and proteomic analyses over Hs 313.T cells in this study due to their biological relevance and technical suitability. HKB-11 is a hybrid cell line derived from HEK293S and 2B8 Burkitt lymphoma cells.

### 2.2. ROS Production in the HKB-11 Lymphoma Cells After Treatment with Different Concentrations of N, UB, and N: UB (7:3)

**Figure 1:**
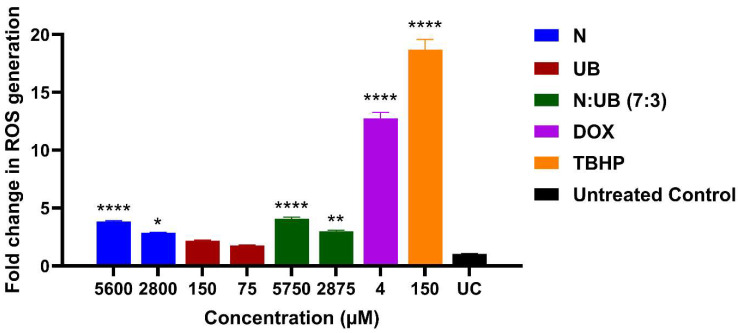
The effect of different concentrations of nisin (N; 5600 μM and 2800 μM), urolithin B (UB; 150 μM and 75 μM), and the combination of N and UB (7:3; 5750 μM and 2875 μM) on the production of reactive oxygen species (ROS) in the HKB-11 lymphoma cell line. For comparative purposes, doxorubicin (Dox) (4 μM) and tert-Butyl hydroperoxide (TBHP; 150 μM) were included as positive controls. The values are expressed as the mean ± SD. * indicates 0.01 < value of *p* < 0.05, ** indicates *p* < 0.01, and **** indicates *p* ≤ 0.0001 compared to the untreated control.

ROS are key mediators of cellular oxidative stress and have been linked to tumour initiation, progression, and metastasis [20]. Figure 1 presents the levels of ROS generated in HKB-11 lymphoma cells following exposure to different concentrations of N (5600 μM and 2800 μM), UB (150 μM and 75 μM), and their combination (7:3; 750 μM and 2875 μM). Dox (4 μM) and tert-Butyl hydroperoxide (TBHP, 150 μM) were positive controls for ROS induction.

N induced a significant, concentration-dependent increase in ROS production compared to the untreated control (*p* < 0.0001 at 5600 μM; *p* < 0.05 at 2800 μM). In contrast, treatment with UB at both concentrations resulted in no significant elevation of ROS (*p* < 0.01 at 2875 μM; *p* < 0.0001 at 5750 μM) compared to the untreated control, suggesting a minimal pro-oxidative effect. This indicated that UB may have a neutral or possibly antioxidant role, consistent with findings that certain compounds help modulate oxidative environments without directly increasing ROS [21]. Furthermore, the N and UB combination at both tested concentrations resulted in a significant increase in ROS levels compared to the untreated control (*p* < 0.01 at 2875 μM; *p* < 0.0001 at 5750 μM), which remained higher than or equal to those induced by N alone (*p* < 0.0001 at 5600 μM; *p* < 0.05 at 2800 μM). Thus, ROS production under the combination treatment was statistically higher than that of the untreated control (*p* < 0.0001 at 5750 μM and *p* < 0.01 at 2875 μM), yet markedly lower than in the positive control groups Dox and TBHP (*p* < 0.0001 at 4 μM; *p* < 0.0001 at 150 μM), respectively. As expected, Dox and TBHP produced the greatest ROS responses in the assay, validating the experimental system as a positive control [18,19,22,23].

Previously, N treatment was found to induce a significant, concentration-dependent increase in ROS production in both MCF7 and MDA-MB-231 breast cancer cells [9,24], suggesting that oxidative stress contributes to the cytotoxic effects in cancer cells [25]. These studies supported the hypothesis that ROS-mediated mechanisms are involved in N-induced apoptosis in breast cancer models. Moreover, several studies have reported similar findings in other types of cancer. For instance, it has been demonstrated that N causes ROS accumulation in gastrointestinal epithelial cells, leading to apoptosis through mitochondrial depolarisation and cytochrome c release [11]. Additionally, Joo et al. (2012) found that N treatment led to the generation of ROS and apoptotic cell death in HeLa cervical cancer cells, suggesting that oxidative stress plays a crucial role in initiating caspase activation and DNA fragmentation [26,27]. Mechanistically, the ROS generated by N may activate multiple apoptotic pathways, including p53 signalling, caspase 3/9 activation, and MAPK cascades such as JNK and p38 [28,29]. Furthermore, these pathways converge to promote apoptosis by inducing DNA damage, cell cycle arrest, and permeabilisation of the mitochondrial outer membrane [28,29]. These findings collectively support the role of ROS induction as a central mechanism in N’s antiproliferative activity, underscoring its potential as a redox-modulating agent in cancer therapy. Finally, our data suggest that while N alone exerts a pro-oxidant action, co-administration with UB attenuates this effect in HKB-11 lymphoma cells. Therefore, the N and UB combination (7:3) induced a controlled ROS response compared to standard chemotherapy Dox, potentially sufficient to trigger apoptosis in lymphoma cells (Figure 1).

### 2.3. Flow Cytometric Analyses of Apoptotic Profiles of Mono and Combination Therapies

**Figure 2:**
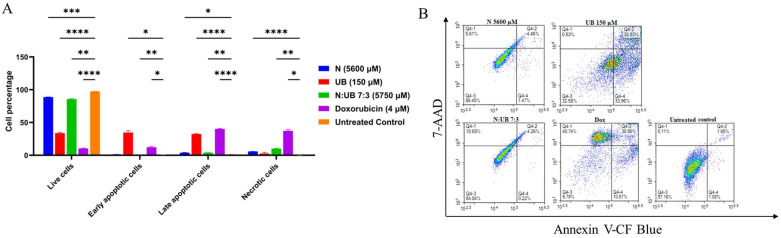
A flow cytometric assessment of the apoptotic profiles of the HKB-11 lymphoma cancer cells after 24 h of treatment. (**A**) The live, early apoptotic, late apoptotic, and necrotic cell percentages after 24 h treatment with nisin (N) (5600 μM), urolithin B (UB) (150 μM), N: UB (7:3; 5750 μM), doxorubicin (Dox) (4 μM), and the untreated control (*n* = 6), respectively. * indicates 0.01 < value of *p* < 0.05; ** indicates *p* < 0.01; *** indicates *p* < 0.001; and **** indicates *p* < 0.0001 compared to the untreated control. (**B**) The density plots of each drug treatment are represented, which are the most representative of the average data from the flow cytometric analyses. Q4-1 indicates necrotic cells, Q4-2 indicates late-stage apoptotic cells, Q4-3 indicates live cells, and Q4-4 indicates early-stage apoptotic cells.

Flow cytometry was used to evaluate apoptotic responses in HKB-11 lymphoma cells after 24 h of exposure to individual and combined N and UB treatments. Figure 2A presents quantitative apoptotic profiles, whereas Figure 2B illustrates the representative density plots.

Individual treatments of N and UB exhibited significant apoptotic induction in HKB-11 lymphoma cells. Specifically, UB at a concentration of 150 μM demonstrated pronounced apoptotic activity, significantly surpassing the untreated control (*p* < 0.0001). This effect highlighted UB’s high potency, even at lower doses, in inducing apoptosis in HKB-11 lymphoma cells. Treatment with N alone moderately increased populations in early (Q4-4) and late apoptosis (Q4-2). In contrast, UB monotherapy dramatically shifted cells toward late apoptosis, substantially reducing viability. The combination N:UB (7:3) exhibited an intermediate profile, indicating partial improvement over N alone but failing to match UB’s superior apoptotic induction. Conversely, N at 5600 μM showed a modest increase in apoptosis. However, it was less effective than UB compared to the N treatment, with a higher proportion of live cells.

The combination treatment of N and UB (7:3; total concentration 5750 μM) significantly enhanced apoptotic induction compared to the untreated control and N alone (*p* < 0.01). However, the apoptotic response generated by this combination was intermediate, indicating that although UB improved N’s apoptotic activity, this combination ratio diluted UB’s inherent effectiveness, leading to a less potent apoptotic effect than UB monotherapy.

Dox (4 μM), employed as a positive control, significantly induced apoptosis (*p* < 0.0001) compared to the untreated control, demonstrating efficacy comparable to the N and UB combination (7:3). Nevertheless, Dox shows similar efficacy to UB alone in the early apoptotic stage. Importantly, necrotic cell populations remained minimal across all postbiotic treatments, unlike Dox, suggesting that apoptosis was the predominant mode of cell death rather than necrosis.

Previous studies support these observations. UB-induced apoptosis has been linked with mitochondrial dysfunction, ROS generation, and activation of apoptotic cascades. For instance, Alzahrani et al. (2021) demonstrated UB’s effectiveness in triggering apoptosis through DNA damage pathways in leukaemia cells [17]. Similarly, UB’s anticancer efficacy via PI3K/Akt and MAPK pathway modulation has been documented, further validating its potent anticancer potential [30,31,32,33]. These mechanisms likely contribute to the robust apoptotic effect of UB observed in HKB-11 cells in the current study. Conversely, N’s modest apoptotic impact aligns with previous findings where higher concentrations were necessary for significant anticancer activity, limiting its standalone effectiveness [26,34].

Dox’s moderate apoptotic effect aligns with its established anticancer mechanisms but faces known resistance challenges, particularly in B-cell lymphomas with BCL-2 overexpression [35]. In contrast, UB’s distinct apoptotic potency might bypass these resistance mechanisms, offering significant clinical advantages. These findings suggested that while the N and UB combination at a 7:3 ratio significantly enhances apoptosis compared to N alone, it did not reach the superior apoptotic potency observed with UB monotherapy. This emphasises the need to optimise the combination ratios further to harness maximal therapeutic efficacy.

Briefly, UB is a potent inducer of apoptosis at low concentrations, primarily through mitochondrial dysfunction, activation of intrinsic apoptotic pathways, and epigenetic modulation [12,13,14,16,17]. In contrast, N predominantly exerts cytotoxicity through membrane disruption and ROS-mediated stress [9,11,24,26,27]. When combined at a fixed 7:3 ratio, the higher total concentration and the strong ROS induction by N may partially overwhelm or attenuate the specific mitochondrial and epigenetic apoptotic pathways triggered by UB, thereby diluting its maximal apoptotic effect. Moreover, this finding is consistent with our proteomic data, which showed the strong suppression of ribosomal proteins and the moderate induction of oxidative stress pathways in the combination group, suggesting that apoptotic signals were distributed across multiple pathways rather than being concentrated on UB’s mitochondrial-driven mechanisms [14,36]. In summary, UB alone (150 µM) exhibited strong apoptotic activity. Furthermore, the N:UB (7:3) combination further augmented the overall cytotoxic effect by significantly reducing viable cells and consistently increasing apoptotic populations compared to the untreated control.

### 2.4. Proteomics Study of the HKB-11 Lymphoma Cells Treated with the Synergistic Combination vs. Mono Treatments

A bottom-up, label-free quantification proteomics analysis was carried out using Micro-UPLC-QTOF-MS/MS, based on a recent protocol developed by our group [9,37,38]. The goal was to identify significant changes in the expression of biomarkers related to apoptosis and cancer development following treatment with the most effective postbiotics and their combinations compared to the untreated control [9,18,37,38]. The HKB-11 lymphoma cells were exposed to N (5600 µM), UB (150 µM), and the N and UB combination (7:3; 5750 µM). Additionally, each treatment group was analysed against the untreated control group to identify changes in the global proteome that could be linked to the antiproliferative effects. Furthermore, comprehensive proteomic profiling of HKB-11 lymphoma cells after each treatment is presented in the attached Supplementary Material File, with Tables S1–S5. Proteins that exhibited differential expression were chosen using stringent statistical criteria of absolute log_2_ FC ≥ 0.58 and Q ≤ 0.05.

#### 2.4.1. Differentially Expressed Proteins (DEPs) in N (5600 µM) Treated HKB-11 Lymphoma Cells Compared to Untreated Control (abs log_2_FC ≥ 0.58 and Q ≤ 0.05)

**Figure 3:**
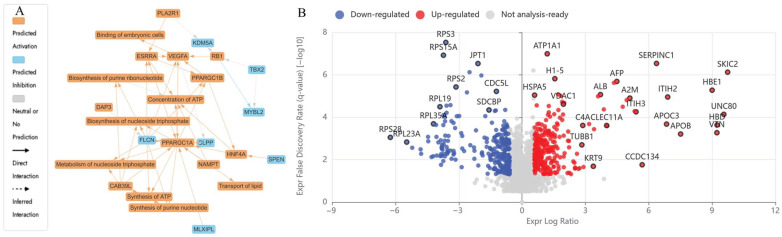
Proteomic profiling of nisin (N)-treated HKB-11 cells reveals distinct molecular responses. (**A**) Ingenuity Pathway Analysis (IPA) graphical summary depicting enriched biological pathways and molecular functions based on significantly regulated proteins following treatment with N (5600 µM). Orange nodes represent predicted activation, blue nodes indicate predicted inhibition, and grey nodes show no prediction. Solid arrows denote direct interactions, while dashed arrows represent inferred interactions. (**B**) Volcano plot showing global changes in protein expression between nisin-treated vs. untreated HKB-11 cells. Red dots indicate significantly upregulated proteins, blue dots indicate significantly downregulated proteins, and grey dots represent non-significant changes or proteins that do not meet analysis thresholds. Significance was determined by absolute log_2_ fold change ≥ 0.58 and Q-value ≤ 0.05. Prominent up- and down-regulated proteins are labelled, highlighting key alterations in response to nisin treatment.

The LCMS-based bottom-up proteomic analysis of HKB-11 lymphoma cell lysate following treatment with N at 5600 µM identified DEPs, which are detailed in Table S1. The Ingenuity Pathway Analysis (IPA) enrichment results are in Table S2 (Supplementary File). Table 2 presents the dysregulated proteins potentially responsible for the antiproliferative activity observed after N treatment. Figure 3 shows the enriched pathways (Figure 3A), the IPA graphical summary (Figure 3B), and the volcano plot comparing N-treated to untreated control cell lysate.

The IPA visual summary presented in Figure 3A illustrates the primary biological insights derived from our IPA core analysis. It highlights and links a selected group of the most statistically significant entities, including canonical pathways, upstream regulators, and biological functions, to create a comprehensive overview. Additionally, by leveraging machine learning, the system ranks these entities and infers potential connections, even in instances where direct links are not yet available in the QIAGEN Knowledge Graph. These inferred relationships contribute to emphasising the broader biological context and interactions identified in the analysis. The IPA summary based on DEPs from the comparison of N versus the control underscores several key biological themes central to the network. Furthermore, the concepts related to cell viability and cell death (a reduction in cell viability, shown in blue in Figure 3A, and the activation of cell death, highlighted in orange in Figure 3A) emerged as a prevalent theme, influenced by regulators such as SKIC2, HBE1, UNC80, VTN, APOC3, APOB, CCDC134, and HBD [39,40,41,42,43]. The upregulation of these proteins converges on oxidative-immune signalling, facilitating cancer cell mortality through redox imbalance, immune activation, and disrupted lipid metabolism.

In the N-treated cells (5600 µM), several proteins involved in redox regulation and mitochondrial function were upregulated. HBA1 was the most highly expressed (Log_2_FC = 8.62), and its elevation has been linked to increased oxidative stress and ferroptosis, contributing to cancer suppression [36,44]. FN1 (Log_2_FC = 1.98), which promotes ECM remodelling and cellular migration in cancer, was also upregulated [45]. In addition, NRAS (Log_2_FC = 0.66) was elevated and has been shown to activate growth inhibition or apoptotic pathways via MAPK and AKT signalling [46]. CYC1 (Log_2_FC = 0.70), a mitochondrial electron transport component, was upregulated and is associated with ROS production and apoptotic induction [47].

Proteins involved in ribosome activity and tumour survival were downregulated upon N treatment at 5600 µM, including DDX21 (Log_2_FC = −1.66) and GNL3 (Log_2_FC = −1.91), which are required for rRNA processing and nucleolar function, primarily by disrupting essential cellular processes such as cell cycle progression and proliferation. By interfering with these functions, the expression of these proteins can lead to cancer cell death (apoptosis) and reduced tumorigenicity [48,49]. PDCD4 (Log_2_FC = −1.04), a known tumour suppressor, and SERBP1 (Log_2_FC = −2.18), involved in mRNA stabilisation, which can inhibit cancer development by affecting mRNA stability and translation, were both reduced [50,51]. Major cell cycle regulators including CCNB1 (Log_2_FC = −0.64), CDK1 (Log_2_FC = −0.79), CKS1B (Log_2_FC = −1.15), UBE2C (Log_2_FC = −1.02), FBXL5 (Log_2_FC = −0.80), TOP2A (Log_2_FC = −0.60), and SKP1 (Log_2_FC = −1.06) were all suppressed, which pointed to mitotic inhibition, disruption of cell cycle progression, induction of cell death (apoptosis), and effects on cancer-promoting pathways [52,53,54,55]. These effects are illustrated in Figure 3A,B.

**Table 2:** The upregulation (red) and downregulation (blue) of the relevant differentially expressed proteins and genes and their associated molecular pathways and mechanisms of action (Q ≤ 0.05) by nisin (N; 5600 µM), urolithin B (UB; 150 µM), and their combination 7:3 (5750 µM) in the HKB-11 lymphoma cell line.

Treatment	Log_2_FC	Gene ID	Protein Descriptions	Molecular Pathway	Mechanism of Action	Reference
N 5600 μM	8.62	*HBA1*	Hemoglobin subunit alpha	Oxidative stress response, heme metabolism, hypoxia-inducible factor-1 (HIF-1) signalling	Upregulation is associated with reduced tumour progression and enhanced oxidative stress, which promotes apoptosis in tumour cells. Upregulation reduces cancer growth via redox disruption and ferroptosis.	[36,44]
	1.98	*FN1*	Fibronectin	Extracellular Matrix (ECM) and integrin signalling	Enhances cell adhesion, migration, epithelial–mesenchymal transition (EMT), and invasion.	[45]
	0.66	*NRAS*	GTPase NRas	RAS/MAPK and PI3K-AKT signalling	NRAS encodes a small GTPase regulating growth signals via MAPK and AKT cascades, inducing apoptosis at high expression.	[46]
	0.70	*CYC1*	Cytochrome c1, heme protein, mitochondrial	Mitochondrial Electron Transport Chain (Complex III)	Involved in oxidative phosphorylation and ROS generation, intrinsic apoptosis.	[47]
	−1.66	*DDX21*	Nucleolar RNA helicase 2	RNA processing	Involved in ribosomal RNA regulation; downregulation supports the inhibition of tumour survival.	[48]
	−1.91	*GNL3*	Guanine nucleotide-binding protein-like 3	Nucleolar stress	Promotes proliferation; downregulation linked to low-grade lymphoma.	[49]
	−1.04	*PDCD4*	Programmed cell death protein 4	PI3K pathway inhibition	Tumour suppressor downregulation facilitates proliferation and chemoresistance.	[50]
	−2.18	*SERBP1*	SERPINE1 mRNA-binding protein 1	mRNA stability	Suppresses tumour suppressor mRNA degradation; downregulation is tumour-permissive.	[51]
	−1.35	*RPSA*	Small ribosomal subunit protein uS2	Cell adhesion	Loss reduces laminin receptor activity and decreases metastatic potential.	[56]
	−0.79	*CDK1*	Cyclin-dependent kinase 1	Cell cycle	Catalyses mitotic onset via phosphorylation of downstream mitotic regulators	[52]
	−1.15	*CKS1B*	Cyclin-dependent kinases regulatory subunit 1	Cell cycle regulation	Binds CDK1/CDK2; regulates p27 degradation	[53]
	−0.80	*FBXL5*	F-box/LRR-repeat protein 5	Iron homeostasis and DNA repair	Degrades iron regulatory proteins; supports replication.	[53]
	−1.06	*SKP1*	S-phase kinase-associated protein 1	Ubiquitin-proteasome pathway	Core component of SCF E3 ligase for cell cycle proteins.	[55]
	−0.60	*TOP2A*	DNA topoisomerase 2-alpha	DNA replication and mitosis	Resolves DNA supercoiling during replication.	[57]
	−1.02	*UBE2C*	Ubiquitin-conjugating enzyme E2 C	Ubiquitination/mitosis exit	Catalyses the degradation of mitotic cyclins.	[54]
UB 150 μM	2.28	*MT-ND2*	NADH-ubiquinone oxidoreductase chain 2	Oxidative phosphorylation	Mitochondrial respiratory chain dysfunction in cancer cells.	[58]
	2.86	*SERPINC1*	Antithrombin-III	Coagulation cascade	Associated with thrombosis in cancer, a potential biomarker for metastasis risk.	[59]
	5.21	*VTN*	Vitronectin	ECM-receptor interaction, FAK/AKT pathway	Promotes metastasis via EMT and integrin signalling.	[42]
	0.64	*CCNB1*	G2/mitotic-specific cyclin-B1	G2/M checkpoint of cell cycle	Forms a complex with CDK1 to drive mitosis.	[60]
	1.26	*CYCS*	Cytochrome c	Intrinsic apoptotic pathway (mitochondria)	Released from mitochondria to activate caspases (Apaf1 → caspase-9 → caspase-3).	[61]
	−0.73	*DNMT1*	DNA (cytosine-5)-methyltransferase 1	Epigenetic regulation	Silencing of tumour suppressors via DNA methylation.	[62]
	−0.68	*G3BP1*	Ras GTPase-activating protein-binding protein 1	Stress response and RNA metabolism	Modulates stress granule formation, inhibits apoptosis under stress.	[63]
	−2.40	*RSL1D1*	Ribosomal L1 domain-containing protein 1	Ribosome biogenesis	Potential suppressor of uncontrolled protein synthesis in cancer.	[64,65]
	−0.93	*CDK4*	Cyclin-dependent kinase 4	Cell cycle (G1-S transition)	Downregulating CDK4 maintains pRB in its active, hypophosphorylated form, which blocks E2F, causing cell cycle arrest.	[66]
	−0.59	*UBA2*	SUMO-activating enzyme subunit 2	SUMOylation pathway	Regulates oncogenic transcription factors and genome stability.	[67]
	−0.68	*CUL1*	Cullin-1	SCF complex (E3 ubiquitin ligase)	Scaffold for E3 ligase that degrades cell cycle regulators.	[68]
	−0.92	*HDAC1*	Histone deacetylase 1	Epigenetic silencing	Deacetylates histones to repress gene expression.	[69]
	−0.87	*RAC1*	Ras-related C3 botulinum toxin substrate 1	Rho GTPase signalling	Controls cytoskeleton, proliferation, and survival.	[70]
	−0.67	*RAC3*	Ras-related C3 botulinum toxin substrate 3	Cell motility and metastasis (GTPase signalling)	Similar to RAC1, with roles in aggressive tumours.	[71]
	−4.23	*RPL27A*	Large ribosomal subunit protein uL15	Ribosome biogenesis, mTOR, p53-MDM2	Protein synthesis, p53 stabilisation via ribosomal stress.	[72]
	−1.04	*RPS14*	Small ribosomal subunit protein uS11	Regulates erythropoiesis and apoptosis	Loss activates p53, which is linked to 5q-syndrome. Mutated in 5q-syndrome (MDS).	[73]
N: UB 7:3 (5600: 150 μM)	−0.87	*RPL5*	Large ribosomal subunit protein uL18	p53-mediated cell cycle pathway	Ribosomal dysfunction, tumorigenesis.	[74]
	−1.48	*RPL11*	Large ribosomal subunit protein uL5	p53-mediated cell cycle pathway	Ribosomal dysfunction, tumorigenesis.	[75]
	−2.03	*RPS19*	Small ribosomal subunit protein eS19	Ribosome biogenesis, possibly p53 pathway	Ribosomal dysfunction.	[76]
	−3.47	*RPS27*	Small ribosomal subunit protein eS27	Ribosome biogenesis/p53 regulation	Suppression leads to reduced ribosomal activity, possibly reducing protein synthesis, but may also destabilise p53, counteracting anticancer effects.	[72]
	−0.60	*UBE2N*	Ubiquitin-conjugating enzyme E2 N	DNA damage response, NF-κB signalling	Downregulation inhibits DNA repair and pro-survival signalling, leading to sensitisation to chemotherapy and apoptosis.	[77]
	0.59	*PSMD10*	26S proteasome non-ATPase regulatory subunit 10	26S proteasome, cell cycle	shRNA targeting PSMD10 significantly reduces the viability of cancer cells. miR-214 and miR-137 inhibit proliferation by suppressing PSMD10.	[78]

#### 2.4.2. DEPs in UB (150 µM) Treated HKB-11 Lymphoma Cells Compared to Untreated Control (abs log_2_FC > 0.58 and Q < 0.05)

**Figure 4:**
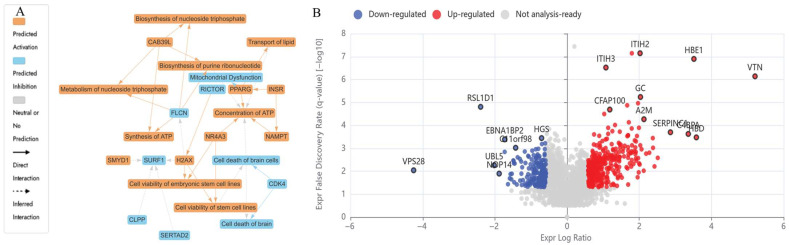
Proteomic analysis reveals cellular pathways modulated by urolithin B (UB) treatment in HKB-11 cells. (**A**) An Ingenuity Pathway Analysis (IPA) graphical summary highlighting two major biologically enriched themes derived from significantly regulated proteins following treatment with UB (150 µM). Coloured nodes represent predicted pathway activity: orange indicates predicted activation, blue indicates predicted inhibition, and grey indicates no prediction. Arrows show relationships—solid for direct and dashed for inferred interactions. (**B**) A volcano plot displaying the distribution of differentially expressed proteins between urolithin B-treated and untreated HKB-11 cells. Red dots represent significantly upregulated proteins, blue dots represent significantly downregulated proteins, and grey dots indicate proteins that did not meet the threshold for significance. Differential expression was defined by an absolute log_2_ fold change ≥ 0.58 and a Q-value ≤ 0.05. Select significantly altered proteins are annotated, illustrating the molecular impact of urolithin B on cellular protein networks.

Table 2 presents the dysregulated proteins in HKB-11 lymphoma cells following 24 h of UB treatment. Figure 4A,B displays the profiles of the dysregulated proteins. The UB treatment at 150 µM upregulated several proteins, including HBA1 (Log_2_FC = 3.69) and CYCS (Log_2_FC = 1.26), both of which are associated with mitochondrial oxidative stress and the activation of the intrinsic apoptotic cascade [43,61]. MT-ND2 (Log_2_FC = 2.28), a mitochondrial respiratory chain component, was upregulated upon UB treatment, consistent with metabolic stress and dysfunction in tumour cells [58]. SERPINC1 (Log_2_FC = 2.86), upregulated in this study, is involved in coagulation balance, potentially inhibits inflammation, and was previously reported to have tumour suppressor effects in certain cancers, including hepatocellular carcinoma (HCC) and potentially colorectal cancer. VTN (Log_2_FC = 5.21), which is related to ECM interaction and the EMT process [42,59], was also increased upon UB treatment.

Proteins involved in epigenetic regulation and translation were downregulated, including DNMT1 (Log_2_FC = −0.73), a DNA methyltransferase, which plays a crucial role in maintaining DNA methylation patterns. Silencing DNMT1 inhibits cancer cell proliferation and suppresses the methylation of tumour suppressor genes, including G3BP1 (Log_2_FC = −0.68), a stress granule component in regulating RNA metabolism. Knockdown of G3BP1 can disrupt stress granule formation and reduce cellular stress responses, potentially leading to the inhibition of cancer cell growth, and RSL1D1 (Log_2_FC = −2.40) is involved in the Wnt signalling pathway, which is often dysregulated in cancer. A reduction in RSL1D1 may inhibit Wnt signalling, leading to a decrease in cancer cell growth, migration, and invasion, all of which are implicated in RNA metabolism and oncogenic stress responses [62,63,64]. Suppression was also observed in cell cycle and ubiquitination regulators, and signalling pathways that promote tumour growth, such as CDK4 (Log_2_FC = −0.93), UBA2, UBE2C, CKS1B, CUL1, and SKP1 [54,66,68,79,80]. Additionally, HDAC1 (Log_2_FC = −0.92), RAC1, and RAC3 were also reduced, suggesting altered epigenetic and cytoskeletal regulation, which may disrupt key signalling pathways that promote cancer growth and spread [69,70,71].

#### 2.4.3. DEPs in Combination 7:3 (5750 µM), Which Are N (5600 µM) and (150 µM), Respectively, Treated HKB-11 Lymphoma Cells Compared to Untreated Control (abs log_2_FC > 0.58 and Q < 0.05)

**Figure 5:**
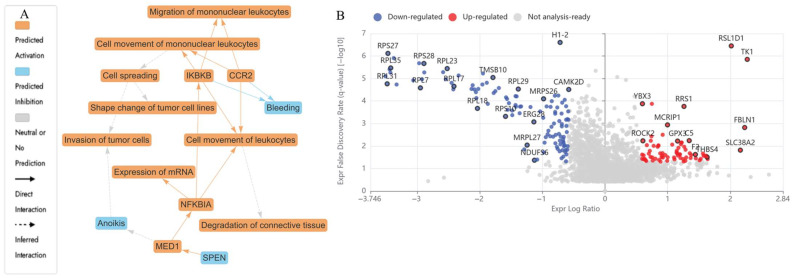
Proteomic profiling of HKB-11 cells reveals distinct molecular responses to the synergistic combination of nisin (N) and urolithin B (UB). (**A**) An Ingenuity Pathway Analysis (IPA) graphical summary illustrating two enriched biological themes arising from significantly modulated proteins following treatment with a synergistic combination of nisin (N) and urolithin B (UB) in a 7:3 ratio (5750 µM total), compared to mono-treatments with N (5600 µM) or UB (150 µM). Nodes represent molecular functions or regulators: orange indicates predicted activation, blue indicates predicted inhibition, and grey denotes neutral or no prediction. Solid and dashed lines indicate direct and inferred molecular interactions, respectively. (**B**) Volcano plot showing differentially expressed proteins unique to the combination treatment versus individual monotherapies. Proteins meeting the significance criteria (absolute log_2_ fold change ≥ 0.58 and Q-value ≤ 0.05) are highlighted: red for upregulated and blue for downregulated proteins. Grey dots indicate proteins not significantly changed. Labelled proteins represent key effectors that may contribute to the enhanced anticancer activity observed with the combination therapy.

In the group treated with the N and UB (7:3) combination, extensive downregulation of ribosomal proteins was observed, as visualised in Figure 5A,B, including RPS14 (Log_2_FC = −1.04), RPL5 (Log_2_FC = −0.87), RPL11 (Log_2_FC = −1.48), RPS19 (Log_2_FC = −2.03), and RPS27 (Log_2_FC = −3.47), all of which are essential for ribosome biogenesis, cell cycle, and tumour suppression through p53 stabilisation [72,73,74,75,76]. UBE2N (Log_2_FC = −0.60), a regulator of DNA repair and NF-κB signalling, was reduced, indicating increased susceptibility to damage. This reduction can hinder cancer growth and survival by altering protein degradation, impacting immune signalling, and potentially reactivating tumour suppressor genes like p53 [77]. PSMD10 (Log_2_FC = 0.59) is involved in proteasomal activity and antiproliferation by interacting with various proteins and pathways, ultimately affecting cell cycle control, apoptosis, and tumour growth. By binding to proteins such as STAT3, Rb, Cdk4, and p53, PSMD10 can modulate signalling pathways, including NF-κB, AKT/mTORC1, and cell cycle checkpoints, ultimately suppressing tumour growth. However, a slight upregulation of PSMD10 was observed, suggesting possible compensation [78].

**Figure 6:**
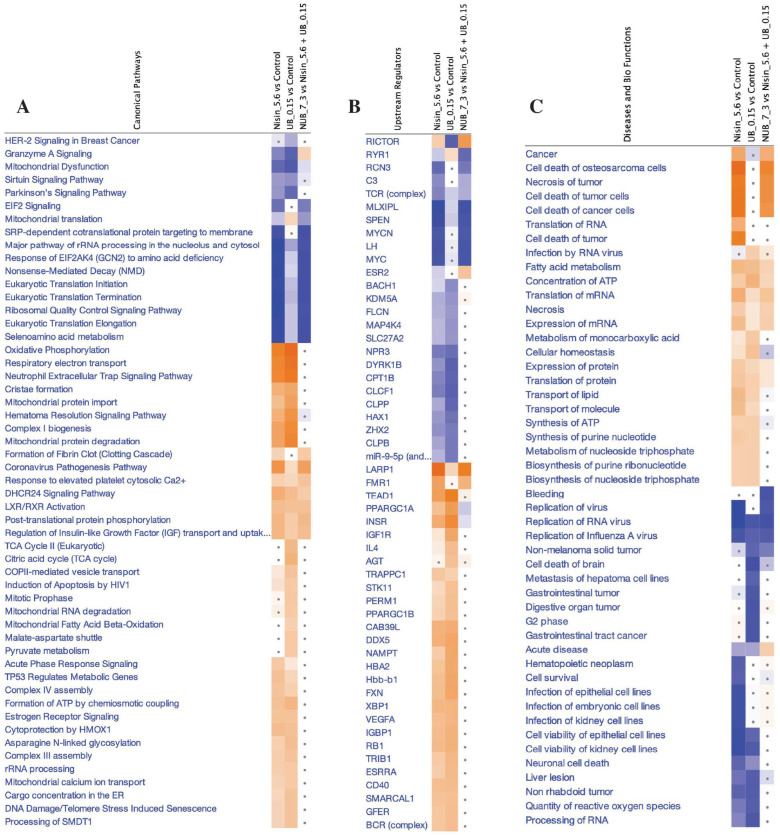
Comparative pathway analysis of proteomic responses to combination vs. monotherapies in HKB-11 cells. Ingenuity Pathway Analysis (IPA) was used to compare significant proteomic changes in HKB-11 lymphoma cells treated with a combination of nisin (N) and urolithin B (UB) (7:3 ratio; 5750 µM) versus monotherapies (N: 5600 µM; UB: 150 µM). Three categories of enrichment are shown: (**A**) Canonical pathways were selected based on Benjamini–Hochberg (BH)-corrected *p* values ≤ 0.001 and an absolute activation Z-score ≥ 2. (**B**) Upstream regulators (genes/proteins) were inferred using the same statistical threshold (*p* ≤ 0.001) and an absolute Z-score ≥ 3. (**C**) Enriched diseases and biological functions were filtered by *p* ≤ 0.001 and an absolute Z-score ≥ 2. The colour intensity represents the activation Z-score of predicted activation (orange) or inhibition (blue), while dotted entries indicate terms with low predicted activation/inhibition (|Z| ≤ 1).

The IPA graphical summary (Figure 5A) highlighted key biological themes, including marked inhibition of ribosome-associated pathways, enhanced apoptosis signalling, and modulation of the DNA damage response in the HKB-11 cells upon treatment with the N and UB combination (7:3). Moreover, canonical pathways analysis (Figure 6A) demonstrated significant disruption in cell cycle regulation, enhanced apoptosis, and suppressed tumour proliferation pathways. Enriched disease and biological functions (Figure 6B) confirmed the reduced metastatic potential and substantial induction of apoptosis, emphasising the combination’s enhanced anticancer effectiveness. Predicted upstream regulators (Figure 6C) indicated potent inhibition of pro-survival and proliferative mediators such as MYC, coupled with activation of stress-response and apoptosis pathways, including TP53 and IFNG.

Collectively, these proteomic findings illustrated a distinct mechanism of the N and UB combination (7:3), characterised by pronounced suppression of ribosomal biogenesis, extensive disruption of cell cycle control, and robust activation of apoptotic and stress-response mechanisms, significantly surpassing the effects of mono-treatment. Thus, these data underscore the potent synergistic potential of this combination therapy in targeting critical tumour survival pathways in lymphoma.

## 3. Materials and Methods

### 3.1. Chemicals and Drug Preparation

Both metabolites used in the study, N and UB, were purchased from Sapphire Bioscience (Redfern, NSW, Australia). Doxorubicin (Dox) was purchased from Sigma Aldrich (Castle Hill, NSW, Australia). Furthermore, all reagents were prepared according to the standard methods and protocols provided with the assay kits.

### 3.2. Cell Culture

Hs 313.T (ATCC CRL-7235), HKB-11 (ATCC CRL-12568; human kidney/B cell hybrid), and HS-5 (ATCC CRL-3611) cell lines were sourced from the American Type Culture Collection (ATCC, Manassas, VA, USA). The Hs 313.T lymphoma cells were cultured in ATCC-formulated Dulbecco’s Modified Eagle’s Medium (DMEM; ATCC 30-2002) containing 4.5 g/L glucose, L-glutamine, and sodium pyruvate. The medium was supplemented with 10% foetal bovine serum (FBS; Bio-Strategy PTY, Campbellfield, VIC, Australia) and 1% penicillin–streptomycin (Sigma Aldrich, Castle Hill, NSW, Australia). The HKB-11 cells were maintained in ATCC-formulated DMEM: F12 (1:1 mixture of DMEM and F-12) supplemented with 10% FBS and 1% penicillin–streptomycin. The HS-5 cells were cultured in the same DMEM formulation used for Hs 313.T, with identical supplements. All cell lines were incubated at 37 °C in a humidified atmosphere with 5% CO_2_. Subculturing was performed every 48 to 72 h to maintain cell confluency.

### 3.3. Cell Viability Assays

Cell viability of HKB-11, Hs313.T, and HS-5 cells following treatment with the combination at 7:3 (5750 μM) was assessed using the Alamar Blue assay, as described by Al-Khazaleh et al. (2025), Alsherbiny et al. (2021), and Dissanayake et al. (2023) [18,38,81]. Briefly, the cells were seeded in 96-well plates at a density of 3 × 10^5^ cells/mL in a 100 μL volume per well. After 24 h, the cells were treated with the postbiotics and then incubated for 72 h. Doxorubicin (Dox; 4 μM; Sigma-Aldrich, Castle Hill, NSW, Australia) was used as a positive control. A vehicle control (0.1% DMSO) was included on each plate.

After the incubation period, the media were removed and replaced with 100 μL of Alamar Blue solution (0.1 mg/mL resazurin), prepared from a 1 mg/mL stock in PBS and diluted 1:10 with serum-free medium. The cells were then incubated for an additional 2.5 h. Fluorescence was measured using a microplate spectrophotometer (BMG CLARIOstar, Mornington, VIC, Australia) with an excitation wavelength of 555 nm and an emission wavelength of 595 nm. Each treatment was conducted in triplicate. Cell viability was then normalised to the untreated control, which was defined as 100%.

### 3.4. Synergy Analysis

As described previously, N and UB were combined to assess their interaction using the combination index (CI) analysis [18]. The CI values were calculated using CompuSyn software version 2.0 (Biosoft, San Ramon, CA, USA), which applies the median-effect equation based on the mass-action law. A six-point dose–response curve was generated for the mono treatments and their combination. The CI model was used to categorise interactions as synergistic (CI < 1), additive (CI = 1), or antagonistic (CI > 1), in line with established methods from our earlier work [38].

### 3.5. Analysis of ROS Production

N, UB, and their combination at 7:3 were studied on the oxidative stress of the cancer cells as per the protocol using the H2DCFDA (2′,7′-dichlorofluorescein diacetate) cellular ROS Detection Assay Kit (#ab113851; Abcam, Melbourne, VIC, Australia) [9,81]. Briefly, HKB-11 lymphoma cells (2.5 × 10^5^ cells/mL) were cultured in a 96-well plate, allowed to adhere overnight, and treated with 20 μM H2DCFDA for 45 min to assess ROS levels. The dye solution was removed, and cells were washed with 1× buffer. Next, the cells were treated with N (5600 and 2800 μM), UB (150 and 75 μM), N: UB (5750 and 2875 μM), Dox (4 μM), and tert-Butyl hydroperoxide (TBHP; 150 μM), and then incubated at 37 °C for 4 h. Finally, the plate was immediately read at Ex/Em = 485/535 nm using a microplate spectrophotometer (BMG CLARIOstar, VIC, Australia). The fold-change in ROS production was determined relative to the untreated control (cells treated with the supplement buffer according to the manufacturer’s protocol).

### 3.6. Flow Cytometry Analyses of the Apoptotic Profiles

The effects of N, UB, and their synergistic combination on the apoptosis profiles of the HKB-11 lymphoma cells after 24 h treatment were studied using an annexin V and 7-AAD-based kit (#ab214663, Abcam, Melbourne, VIC, Australia) [9,81]. The HKB-11 cells were cultured in T75 cell culture flasks at an initial density of 3 × 10^6^ cells per 10 mL at 37 °C in the presence of 5% CO_2_ for 24 h. The following day, the cell culture media was removed from each flask and replaced with fresh media containing FBS. The cultured flasks were then treated with the highest concentration of the most active postbiotics (5600 μM) N, (150 μM) UB, and the positive control Dox (4 μM). A serum-containing medium was used as the untreated control. The flasks were then incubated at 37 °C with 5% CO_2_ for 24 h. Then, the cell culture media from each flask were collected. Subsequently, trypsin (0.25% *w*/*v*) was added to the flasks and incubated for 4 min at 37 °C. The trypsin reaction was neutralised with an equal volume of 10% FBS serum-containing media, and the cells were combined with the previously collected media. The cell pellets were obtained by centrifuging at 500× *g* for 5 min at room temperature (RT). This procedure was repeated by suspending the cell pellets in 1 mL of PBS each time. The collected cell pellets from each treatment were immediately suspended in 500 μL of 1× binding buffer and gently mixed by pipetting. Annexin V-CF blue (5 μL) and 7-AAD (5 μL) staining solutions were added to 100 μL cell suspension. The stained cells were incubated for 15 min in the dark at RT, after which 400 μL of a 1× assay buffer was added to each cell suspension. Subsequently, the cells were examined using a flow cytometer (Novocyte 3000, ACEA Biosciences Inc., San Diego, CA, USA), and data analysis and processing were performed using the NovoExpress software (version 1.5.0, ACEA Biosciences Inc., CA, USA). In the initial step, the cells were gated on forward and side scatter modes to exclude cell aggregates and debris near the origin. The cells were then gated on dot plots, where Annexin V-CF in Pacific Blue was plotted against 7-AAD fluorescence in PerCP. Quadrants were positioned relative to the untreated control, indicating live cells (+Annexin V and −7-AAD) appearing in the lower-left quadrant, early apoptotic cells (+Annexin V and −7-AAD) in the lower-right quadrant, late apoptotic cells (+Annexin V and +7-AAD) in the upper-right quadrant, and necrotic cells (−Annexin V and +7-AAD) in the upper-left quadrant. For statistical analyses and visualisation, the percentage data of cells in each quadrant after different treatments (*n* = 3) were exported to GraphPad Prism software (version 9.0, San Diego, CA, USA).

### 3.7. Liquid Chromatography–Mass Spectrometry (LC–MS)–Driven Bottom-Up Proteomics Analysis

#### 3.7.1. Cell Culture, Treatment, and Protein Extraction

Lymphoma cells HKB-11 were placed in T75 flasks at a concentration of 3.0 × 10^5^ cells/mL and incubated overnight at 37 °C in 5% CO_2_. After removing the media, fresh DMEM/F-12 medium supplemented with 10% FBS was added, and the cultured flasks were treated with specific doses of N (5600 μM), UB (150 μM), and their combination (7:3, 5750 μM). Treatments were performed in triplicate and incubated for 24 h under the same conditions. Following incubation, each flask of cells was subjected to a 0.25% *w*/*v* trypsin treatment for 4 min at 37 °C, and the cell culture medium was collected. Additionally, an equal volume of DMEM F-12 medium (containing 10% FBS) was added before mixing with the previously collected media to neutralise the trypsin. The cells were spun in a centrifuge at 500× *g* for 5 min at RT. The cell pellets were washed twice with ice-cold PBS and spun again at 500× *g* for 5 min. These cell pellets were then suspended in a lysis buffer that included 1 μL of universal nuclease and a Halt™ Protease and Phosphatase Inhibitor Cocktail (Thermo Fisher Scientific, Sydney, NSW, Australia), which was fully compatible with mass spectrometry (MS). The cells were gently pipetted 10–15 times to reduce the sample’s viscosity and then placed on ice for 20 min. The lysate was centrifuged at 14,000 rpm for 20 min at 4 °C, and the resulting liquid was collected.

#### 3.7.2. Protein Quantification

Pierce™ Rapid Gold BCA Protein Assay Kit (#A53226, Thermo Fisher Scientific, Sydney, NSW, Australia) was used to determine the protein concentration of the cell lysate in triplicate using a bovine serum albumin (BSA) standard, following the manufacturer’s protocol [9,37]. In brief, 1 μL of each sample replicate was diluted 1:20 in Milli-Q water, along with 20 μL of each standard, and then placed in a 96-well plate, with 200 μL of working reagent in each well. Samples were diluted to a concentration within the 20–2000 μg/mL working range. The plate was thoroughly mixed on a plate shaker for 30 s, incubated at RT for 5 min, and then the absorbance was measured within 20 min at 480 nm using a microplate spectrophotometer (BMG CLARIOstar, Melbourne, VIC, Australia). The blank absorbance was subtracted from all other readings of standards and samples, and the sample concentration was determined using the established BSA standard calibration curve. The samples were then stored at −80 °C for further analysis.

#### 3.7.3. Peptides Preparation and Clean-Up

Protein samples (100 μg) were subjected to chemical and enzymatic sample processing using the EasyPep™ Mini MS Sample Prep Kit following the manufacturer’s instructions (Thermo Fisher Scientific, Sydney, NSW, Australia) and as reported in the literature [9,37]. Briefly, the sample volume was adjusted to 100 μL using a lysis buffer in a microcentrifuge tube. Subsequently, the reduction and alkylation solutions (50 μL each) were introduced, gently mixed, and incubated at 95 °C with a heat block for 10 min. The samples were allowed to cool to RT, after which 50 μL of the reconstituted trypsin/lys-C protease mixture was added to each sample and incubated with shaking at 37 °C for 3 h. Following incubation, 50 μL of a digestion stop solution was gently mixed into the samples. Peptide clean-up columns were used to remove both hydrophilic and hydrophobic impurities. The resulting clean peptide samples were dehydrated using a vacuum centrifuge and reconstituted in 100 μL of a 0.1% formic acid solution in water for LC–MS analysis. Subsequently, these samples were carefully transferred to maximum recovery sample vials (Waters Corp., Milford, MA, USA).

#### 3.7.4. Label-Free Quantitative Proteomics Using Micro-High-Performance Liquid Chromatography Coupled with Quadruple Time-of-Flight Mass Spectrometry (Micro-HPLC-QTOF-MS)

##### Liquid Chromatography and Mass Spectrometry Setup

Label-free, bottom-up proteomic quantification was performed using a micro-high-performance liquid chromatography system (Waters M-Class) coupled with a SCIEX™ Triple TOF^®^ 6600 quadrupole time-of-flight mass spectrometer, operated in the positive electrospray ionisation mode (ESI+). A total of 4 µg of tryptic peptide digest was injected onto a nanoEase M/Z HSS T3 column (1.8 µm, 300 µm × 150 mm; Waters, Milford, MA, USA, 186009249) with an in-line Zorbax 300SB-C18 guard column (5 µm, 5 × 0.3 mm; Agilent Technologies, Santa Clara, CA, USA). The column temperature was maintained at 40 °C. Mobile phase A consisted of 98% water and 2% acetonitrile, and mobile phase B consisted of acetonitrile with 0.1% formic acid. The system operated at a 5 µL/min flow rate, with loading and column washing steps conducted at 7 µL/min. The LC gradient was as follows: 2–10% B over 1.66 min at 7 µL/min, 10–25% B from 1.67 to 21.67 min at 5 µL/min, followed by a sharp increase to 95% B from 23.33 to 24.67 min, which was held for 2 min and re-equilibrated at 2% B for 9 min at 7 µL/min.

##### Mass Spectrometry Acquisition Parameters

The mass spectrometer featured a DuoSpray ion source and a 25 μM internal diameter electrode. Data were acquired using the Analyst 1.8.1 software suite and associated LC control drivers. The key ion source parameters were as follows: GS1 = 25, GS2 = 15, curtain gas = 20, ion spray voltage floating = 5500 V, and ion source temperature = 150 °C. The acquisition employed the SWATH data-independent acquisition (DIA) strategy, comprising an MS1 survey scan (*m*/*z* 350–1250, 50 ms accumulation) followed by 40 variable-width MS2 windows (*m*/*z* 400–1250), each with a 35 ms accumulation time, covering the full precursor *m*/*z* range. MS2 spectra were acquired in high-resolution mode across *m*/*z* 100–2000, with an overall cycle time of approximately 1.5 s.

##### Mass Calibration and Library Generation

The PepCalMix calibrant (SCIEX, P/N 5045759; 10 fmol/µL), diluted 1:100 in 5% acetic acid and 2% acetonitrile, was injected every 12 samples to ensure mass accuracy. Six pooled quality control (QC) samples were used to construct a DIA-only spectral library using a gas-phase fractionation approach [82], covering *m*/*z* segments of 400–500, 500–600, 600–700, 700–800, 800–900, and 900–1000. The precursor isolation window was set to 5 *m*/*z*, with a collision energy spread of 5 eV, except for *m*/*z* 700–990 (8 eV) and 990–1000 (10 eV). Each DIA segment cycle time was 2.14 s, incorporating both low- and high-energy scans with a 40 ms MS2 accumulation.

##### Data Processing and Statistical Analysis

The data were processed using Spectronaut v19.5, which implemented the DirectDIA+ workflow with the Biognosys Standard (BGS) analysis framework. The canonical human reference proteome (UniProt, released 24 January 2024; 17,179 entries) was used as the reference database. Searches were conducted using Pulsar, with enzymatic specificity for trypsin/P and LysC/P, allowing up to 2 missed cleavages. Peptide lengths were restricted to 7–52 amino acids. Carbamidomethylation (C) was set as a fixed modification, while variable modifications included protein N-terminal acetylation, methionine oxidation, and methylation and demethylation. A maximum of five variable modifications per peptide was permitted. Peptide–spectrum matches (PSMs), peptides, and protein groups were filtered at a% false discovery rate (FDR) of 1%. Label-free quantification (LFQ) was performed automatically using MS2 area integration with default normalisation strategies. Protein inference employed the IDPicker algorithm.

Pathway analyses were performed using various tools, including STRING [83], Reactome [84], g:Profiler [85], and IMPaLA [86], all of which are identify pathways responsible for the synergistic effects against the HKB-11 lymphoma cells.

##### Data Availability

The mass spectrometry proteomics data were deposited in the ProteomeXchange Consortium via the PRIDE [87] partner repository with the dataset identifier PXD063952.

### 3.8. Statistical Analysis

Data were collected and organised using Microsoft Excel and analysed with GraphPad Prism. All experiments were performed in triplicate, and the results are expressed as the mean ± standard deviation. Statistical significance was defined as *p* < 0.05 using two-way ANOVA. Tukey and Dunnett’s post-hoc tests were applied for multiple comparisons and nonlinear regression within GraphPad Prism. IC_50_ values, indicating the concentration required to inhibit 50% cell growth, were calculated using the same software.

For the proteomics analysis, unpaired *t*-tests were used to compare experimental groups, assuming equal variances. Proteins were considered differentially expressed if they met the following criteria: an absolute log_2_ fold change ≥ 0.58 and a Q-value ≤ 0.05. Functional enrichment analysis was performed using Ingenuity Pathway Analysis (IPA), with pathways selected based on an adjusted Q-value ≤ 0.05 and an absolute z-score ≥ 1.

## 4. Conclusions, Limitations, and Future Directions

This study evaluated the anticancer effects of the gut microbiota-derived N and UB combination 7:3 (5750 μM) against lymphoma cells using a stepwise approach that included cytotoxicity screening, oxidative stress analysis, apoptotic profiling, and proteomic characterisation.

The initial cytotoxicity assessment using the Alamar Blue assay demonstrated potent, dose-dependent growth inhibition in two human lymphoma cell lines, HKB-11 and Hs 313.T. The N:UB (7:3) combination exhibited the highest potency, with an IC_50_ of 332.2 µM in Hs 313.T and 820 µM in HKB-11 cells. The combination did not exceed UB’s cytotoxic effects but improved the efficacy of N monotherapy. Importantly, it showed minimal toxicity toward normal stromal cells (HS-5), supporting a favourable therapeutic index.

ROS assays revealed a clear treatment-dependent pattern. N treatment significantly elevated ROS levels in HKB-11 cells in a concentration-dependent manner, suggesting ROS-mediated cytotoxicity as a key mechanism. UB treatment, however, did not induce a significant ROS response, aligning with its known antioxidant or redox-neutral properties. The N:UB (7:3) combination induced moderate ROS production, lower than N alone, but significantly higher than the untreated control, indicating that UB may attenuate N-induced oxidative stress while retaining antiproliferative efficacy.

Flow cytometry analysis using annexin V/7-AAD staining identified UB (150 µM) as the most potent inducer of apoptosis among all treatments. It significantly increased early and late apoptotic populations in HKB-11 cells, surpassing N (5600 µM) and the N:UB combination (7:3). The combination showed enhanced apoptotic effects over N alone but did not match the potency of UB monotherapy. Dox, used as a positive control, confirmed the reliability of the assay. Notably, all treatments exhibited low necrotic activity, except for Dox, suggesting that apoptosis was the primary mode of cell death.

Proteomics analysis, employing label-free quantification and LC-MS/MS, revealed distinct molecular effects for each treatment. N treatment resulted in significant upregulation of proteins linked to oxidative stress (HBA1, FN1, and CYC1) and downregulation of cell cycle regulators (CDK1, CCNB1, and TOP2A), indicating mitochondrial involvement and mitotic suppression. UB treatment led to mitochondrial reprogramming through the upregulation of CYCS, MT-ND2, and VTN, while concurrently downregulating CDK4, DNMT1, HDAC1, and other cell cycle and epigenetic regulators, reinforcing its apoptotic mechanisms. The N:UB combination (7:3) strongly suppressed ribosomal proteins (RPS14, RPL11, and RPS19), suggesting inhibition of ribosome biogenesis and disruption of p53 signalling.

These findings provide novel insights into the molecular interactions of these two postbiotics and support their potential as a synergistic, less toxic therapeutic approach for lymphoma. However, this study has certain limitations: (a) the high in vitro concentrations required for efficacy may not reflect physiologically achievable doses in vivo, (b) the work was restricted to cell line models without animal validation, and (c) the mechanistic findings are primarily correlative and based on proteomic analyses without orthogonal functional validation. Future work should focus on optimising the combination ratio, exploring lower and more clinically relevant dosing strategies, understanding their potential interactions with standard chemo and immunotherapies, and validating the efficacy and safety in appropriate in vivo as well as organoid lymphoma models to inform clinical translation.

## Figures and Tables

**Table 1:** Cell growth inhibition (%) against HKB-11 and Hs 313.T lymphoma cell lines and cell viability (%) of HS-5 normal stromal cell line at different concentrations of nisin (N) and urolithin B (UB) in combination (7:3).

Concentration (μM) N:UB 7:3	Cell Growth Inhibition (%)		Cell Viability (%)
	HKB-11	Hs 313.T	HS-5
5750 (5600:150)	100.16 ± 0.02 ^a^	100.45 ± 0.11 ^a^	1.08 ± 1.43
2875 (2800:75)	100.11 ± 0.03 ^a^	99.05 ± 1.16 ^a^	10.71 ± 1.96
1437.5 (1400:37.5)	80.27 ± 3.41 ^a^	89.25 ± 0.71 ^b^	28.11 ± 5.36
718.75 (700:18.75)	49.15 ± 7.03 ^a^	82.71 ± 0.88 ^b^	72.96 ± 9.29
359.38 (350:9.375)	14.40 ± 5.52 ^a^	56.49 ± 3.83 ^b^	90.69 ± 7.06
179.69 (175:4.6875)	8.53 ± 3.03 ^a^	21.35 ± 1.53 ^b^	93.63 ± 6.49
IC_50_	820 μM	332.2 μM	1091 μM

Data are presented as the mean ± standard deviation (SD). ^a^, ^b^ values in the same row that do not have the same superscript letter are significantly different (*p* < 0.05) from each other.

## Data Availability

The mass spectrometry proteomics data have been deposited in the Proteo-meXchange Consortium via the PRIDE [87] partner repository with the dataset identifier PXD063952.

## Supplementary Materials

The following supporting information can be downloaded at https://www.mdpi.com/article/10.3390/ijms26157369/s1.
